# α-Fe_2_O_3_@Pt heterostructure particles to enable sonodynamic therapy with self-supplied O_2_ and imaging-guidance

**DOI:** 10.1186/s12951-021-01105-x

**Published:** 2021-11-04

**Authors:** Tian Zhang, Qiang Zheng, Yike Fu, Congkun Xie, Gonglin Fan, Yifan Wang, Yongjun Wu, Xiujun Cai, Gaorong Han, Xiang Li

**Affiliations:** 1 State Key Laboratory of Silicon Materials, School of Materials Science and Engineering, Zhejiang University, Hangzhou, 310027 Zhejiang P. R. China; 2 Key Laboratory of Endoscopic Technique Research of Zhejiang Province, Sir Run Run Shaw Hospital, Zhejiang University, Hangzhou, 215123 P. R. China; 3 ZJU-Hangzhou Global Scientific and Technological Innovation Center, Zhejiang University, Hangzhou, 311200 P.R. China

**Keywords:** Heterostructure, Sonodynamic therapy, Self-supplied oxygen, Tumor theranostic

## Abstract

Sonodynamic therapy (SDT), presenting spatial and temporal control of ROS generation triggered by ultrasound field, has attracted considerable attention in tumor treatment. However, its therapeutic efficacy is severely hindered by the intrinsic hypoxia of solid tumor and the lack of smart design in material band structure. Here in study, fine α-Fe_2_O_3_ nanoparticles armored with Pt nanocrystals (α-Fe_2_O_3_@Pt) was investigated as an alternative SDT agent with ingenious bandgap and structural design. The Schottky barrier, due to its unique heterostructure, suppresses the recombination of sono-induced electrons and holes, enabling superior ROS generation. More importantly, the composite nanoparticles may effectively trigger a reoxygenation phenomenon to supply sufficient content of oxygen, favoring the ROS induction under the hypoxic condition and its extra role played for ultrasound imaging. In consequence, α-Fe_2_O_3_@Pt appears to enable effective tumor inhibition with imaging guidance, both in vitro and in vivo. This study has therefore demonstrated a highly potential platform for ultrasound-driven tumor theranostic, which may spark a series of further explorations in therapeutic systems with more rational material design.

**Supplementary Information:**

The online version contains supplementary material available at 10.1186/s12951-021-01105-x.

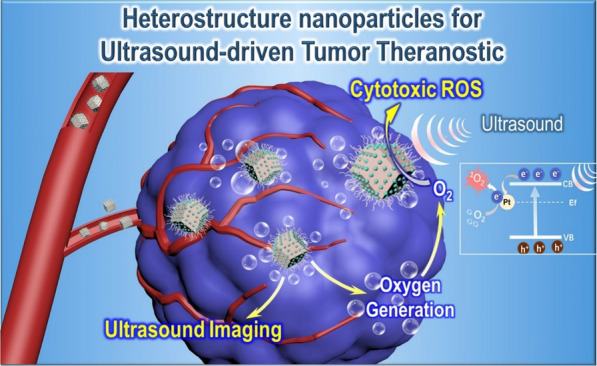

## Introduction

Reactive oxygen species (ROS), comprising singlet oxygen (^1^O_2_), superoxide radicals (O_2_^·−^) and hydroxyl radicals (•OH), are highly active radicals utilized in the investigation of advanced nanomedicine [1]. Therapeutic strategies, boosting ROS generation to induce irreversible damage on liposome and DNA by oxidation, have gained considerable success in tumor treatment in recent years [2, 3]. In the current selections, sonodynamic therapy (SDT) shows significant potential due to the superior penetration depth of ultrasound in tissue (over ~ 10 cm) [4], minimally invasive modality and effective controllability both in spatial and temporal [5, 6]. Owing to the sonoluminescence and pyrolysis phenomena induced by ultrasound cavitation effect, sonosensitizers generate ROS under the activation of certain ultrasound [7, 8]. Despite to the current platforms, such as TiO_2_, ZnO, MnWO_x_ and DHMN [9–12], the design and construction of smarter SDT systems are being endeavored extensively aiming at promoted ROS induction, sensitivity, functional integration and enhanced tumor inhibition.

Considering the core mechanism of sonoluminescence in SDT, ultrasound (US) presents similar characteristics to light spectra with varied wavelengths which may activate specific semiconductors [13, 14]. Under the exposure to ultrasound irradiation, the semiconductors can absorb energy and transfer it to activators, and in turn the electrons on valance band (VB) are excited to conduction band (CB) [15–17]. The electrons migrate to the surface of materials, enabling the redox reaction which converts adsorbed O_2_ into toxic ^1^O_2_ [5]. Due to the crucial role that electrons play in this phenomenon, the semiconductors with appropriate band gap structure, favoring the separation of electrons and holes, are highly demanded. In general, the construction of heterostructure is evidently imperative to promote the quantum yield [15]. When the semiconductor material or noble metals are coupled with the base semiconductor, they may form a heterostructure and induce the spatial charge separation by transferring electrons between two components. The effective spatial separation of sono-generated electrons and holes can be therefore achieved. It is therefore considered that coupling semiconductors with appropriate gaps and Fermi levels is vital for the investigation of advanced sonosensitizers [18]. Typical n-type semiconductor hematite (α-Fe_2_O_3_) with a narrow ban-gap of 2.0–2.2 eV is known as a stable, cost effective and non-toxic catalyst [19]. Owing to its favorable light absorption, it has been extensively explored for the applications in water splitting, sensors and catalysis. Nevertheless, the poor electrical conductivity, low charge transfer efficiency and high electron–hole recombination loss significantly hinder the application of α-Fe_2_O_3_ in SDT [20]. One potential approach to tackle this challenge is to combine hematite with noble metals (e.g. Pt, Au, Pd) [21] with different Fermi levels and certain nanozyme activity. The capture trap effect induces the redistribution of electrons from the conduction band of α-Fe_2_O_3_ to the noble metals. The construction of Schottky barrier at the interface of the pairing may effectively prevent the recombination of ultrasound-induced electrons and holes [9, 22], facilitating the ROS induction and tumor inhibition in SDT.

Meanwhile, O_2_ is transformed into ^1^O_2_ by SDT agents under US irradiation, suggesting the local content of oxygen plays an important role in the ROS induction. The intrinsic hypoxic condition in solid tumors is therefore another severe challenge in achieving the expected outcomes of SDT [23]. In addition, the consumption of oxygen in SDT process exacerbates the tumor hypoxia, further weakening the SDT efficacy [9]. The reoxygenation strategies have been widely utilized in the development of therapeutic systems, including the integration of oxygen reservoirs (e.g. hemoglobin and perfluorocarbon) [24, 25] or catalase-mimicking nanozymes (e.g. MnO_2_ and Pt) [2, 23, 26]. In addition to the supply of oxygen, the integration of O_2_-producing compound may also endow the SDT agents with ultrasound imaging properties according to the pulse-echo principle, enabling tackling the restriction of poor soft tissue contrast and real-time US imaging [27, 28]. Therefore, the pairing of α-Fe_2_O_3_ and Pt appears to be a highly potential concept for constructing the multifunctional SDT system showing promoted ROS induction, self-supplied oxygen and US imaging.

**Fig. 1:**
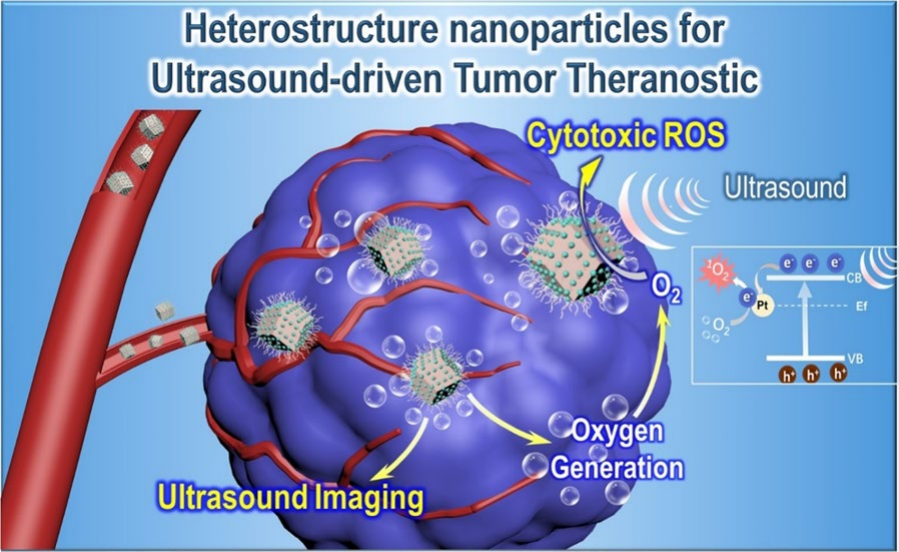
Schematic illustration for the functioning mechanism of α-Fe_2_O_3_@Pt nanoparticles

In this study, for the first time, fine α-Fe_2_O_3_ nanoparticles armored with Pt nanocrystals (α-Fe_2_O_3_@Pt) was designed and synthesized as an alternative SDT agent presenting ingenious bandgap and structural design (Fig. 1). α-Fe_2_O_3_ nanoparticles were prepared by a one-pot hydrothermal method [29], and Pt nanocrystals were integrated on the surface of α-Fe_2_O_3_ to form the heterojunction [30]. The findings indicate that α-Fe_2_O_3_@Pt heterostructure nanoparticles can induce large amounts of singlet oxygen (^1^O_2_) under ultrasound irradiation due to the effective separation of US-induced electrons and holes. More interestingly, Pt nanocrystals with catalase-like activity effectively supply high content of oxygen to modulate tumor hypoxia, and the gas bubbles favor ultrasound imaging purposes. In consequence, α-Fe_2_O_3_@Pt appears to enable effective tumor inhibition with imaging guidance, both in vitro and in vivo. This study has therefore demonstrated a highly potential platform for ultrasound-driven tumor theranostic, and this concept may inspire a series of further explorations in therapeutic systems with more rational material design.

## Materials and methods

### Chemicals and agents

Ferric chloride hexahydrate (FeCl_3_·6H_2_O), sodium dihydrogen phosphate dehydrate (NaH_2_PO_4_), sodium chloride (NaCl), sodium borohydride (NaBH_4_) and 30% hydrogen peroxide aqueous solution (H_2_O_2_) were purchased from Sinopharm Chemical Reagent Co., Ltd. mPEG-SH was obtained from Macklin. Singlet oxygen sensor green (SOSG) and CCK-8 were obtained from Dalian Meilun Biotechnology Co. Ltd. Chloroplatinic acid hexahydrate (H_2_PtCl_6_·6H_2_O) and 1, 3-Diphenylisobenzofuran (DPBF) were purchased from Aladdin Co., Ltd. 2′,7′-Dichlorodihydrofluorescein diacetate (DCFH-DA, ≥ 97%) was purchased from Sigma-Aldrich. All regents were of analytical grade and used as received.

### Characterization

The microstructure of the samples was examined by field-emission scanning electron microscopy (FESEM, Phenom Pharo) and transmission electron microscopy (TEM, Tecnai F20, FEI and Talos F200X). The phase structure was identified by powder X-ray diffraction instrument (XRD, X’pert PRO MPD, scanning 2θ from 10° to 80°). and the X-ray photoelectron spectroscopy (XPS) was characterized on AXIS Supra, Kratos. A zetasizer (Zetasizer Nano-ZS, Malvern) was used to determine the hydrodynamic size by dynamic light scattering (DLS). The UV–vis spectra were measured by TU-1810 UV–vis spectrophotometer and the Fourier transformed infrared (FTIR) spectra were collected by FTIR spectrophotometer (Tensor 27, Bruker). The concentrations of Fe and Pt in the FP solution were determined by inductively coupled plasma optical emission spectrometer (ICP-OES).

### ***Synthesis of ***α***-Fe***_***2***_***O***_***3***_***@Pt heterostructure particles***

α-Fe_2_O_3_ was synthesized by a modified one-pot hydrothermal method [29]. Briefly, 142.8 mg of FeCl_3_·6H_2_O (0.02 M), 0.179 mg of NaH_2_PO_4_ (0.05 mM) and 2.5 mg of NaCl (1.1 mM) were dissolved in 30 mL of ultrapure water and stirred for 10 min at room temperature. Subsequently, the mixture was transferred into a 50 mL Teflon-liner autoclave and heated at 220 ℃ for 5 h. After cooling down to room temperature, the as-prepared brick red product was centrifuged (12,000 rpm, 10 min) for three times, washed with water and ethanol and resuspended in 30 mL of water.

To synthesize α-Fe_2_O_3_@Pt particles, 5 mL of as-prepared α-Fe_2_O_3_ was dispersed in 50 mL of ultrapure water followed by the slow addition of 100 μL H_2_PtCl_6_ solution (20 mg mL^−1^). After vigorously stirring for 20 min, 600 μL of the as-prepared icy NaBH_4_ (0.1 M) aqueous solution was added dropwise. The solution was then centrifuged for three times and concentrated to 10 mL. The prepared α-Fe_2_O_3_@Pt solution was mixed with 80 mg of mPEG-SH. After sonicated for 6 min, the mixture was stirred for two days and subsequently washed using water for three times.

### ***Functional characteristics of ***α***-Fe***_***2***_***O***_***3***_***@Pt***

The catalase-like performance of α-Fe_2_O_3_@Pt hybrids was investigated by measuring the variation of O_2_ concentration with a dissolved oxygen meter in the presence of H_2_O_2_. Briefly, H_2_O_2_ with different concentrations (200 μM, 500 μM, 1 mM,) was mixed with FP solution (50 μg mL^−1^) and the dissolved O_2_ concentration was monitored every 10 s.

A typical ROS detection reagent DPBF was used to detect ROS generation from α-Fe_2_O_3_@Pt under US irradiation. 100 μL of DPBF (2 mM) was injected into the FP solution (50 μg mL^−1^), and then the mixture was exposed to US (1.0 MHZ, 1.0 W cm^−2^) in the dark. The degradation of DPBF was analyzed by the detection of UV–vis absorption spectra every 2 min. The properties of ROS generation from Fe_2_O_3_ and FP + H_2_O_2_ (200 μM) induced by US irradiation were studied in the same way.

A typical ^1^O_2_ molecular probe SOSG was used to detect ^1^O_2_ generation. Briefly, 40 μL of SOSG solution was added into the FP solution (50 μg mL^−1^), and then the mixture was exposed to US (1.0 MHZ, 1.0 W cm^−2^) in the dark. The absorption of SOSG was detected by a fluorescence spectrophotometer every 4 min. In addition, ^1^O_2_ generation of FP solution mixed with H_2_O_2_ (200 μM) was also detected in the same way.

The diffuse reflectance spectroscopy was used to measure the contrast of the UV–vis spectra and the bandgap of Fe_2_O_3_ and FP NPs. The bandgaps were acquired by Tauc plot as the following:

$$(\upalpha h\nu )^{{\text{n}}} = {\text{ B}}(h\upnu - {\text{Eg}})$$

where α is the absorbance at a wavelength λ; hν is photon energy and is equal to hc/λ; c is speed of light; h is equal to 4.1356676969 × 10^–15^ eV‧s; B is a constant; Eg is the bandgap of the semiconductor; n = 2 or 1/2, which is linked to the direct bandgap or indirect bandgap of the semiconductor, respectively. In view of the known indirect bandgap of Fe_2_O_3_, the bandgaps of Fe_2_O_3_ and FP NPs were estimated by a related curve of (αhν)^1/2^ versus photo energy plotted.

Electrochemical Impedance Spectra (EIS) were tested on a conventional three-electrode cell in 0.5 M KHCO_3_ solution using a CHI 760E electrochemistry workstation and in the frequency from 100 kHz to 1 Hz with an AC voltage amplitude of 5 mV. The working electrode was prepared by dropping Fe_2_O_3_ and FP aqueous solution onto a cleaned carbon paper and then dried at 60 ℃ overnight in air. The counter electrode was Pt and the reference electrode was standard Ag/AgCl electrode, respectively.

### In vitro study

4T1 murine breast cancer cells were utilized for in vitro study of anticancer effect. The cells were cultured in RPMI 1640 medium with 10% fetal bovine serum (FBS) in an incubator containing 5% CO_2_ at 37 °C. The hypoxic conditions were carried out in the hypoxic chamber with 2% O_2_.

To investigate the in vitro SDT effect of α-Fe_2_O_3_@Pt, 4T1 cells were cultured in a 35 mm well with the density of 2 × 10^4^ cells mL^−1^ and grown overnight. Then, α-Fe_2_O_3_ and α-Fe_2_O_3_@Pt with different Fe concentrations (0–200 ppm) were added. After incubation for another 4 h, the wells were treated with US irradiation (1.0 MHZ, 1.0 W cm^−2^, 30 s). Then the cells were incubated for another 24 h and finally the relative cell viability was tested by CCK 8 assay. The absorbance was measured by a microplate reader at the wavelength of 450 nm. The SDT effect was studied in normoxia and hypoxia conditions, respectively.

DCFH-DA probe was utilized to detect ROS generation in vitro. Briefly, after seeded for 12–16 h, the cells were mixed with different solutions (control, Pt NPs, α-Fe_2_O_3_, α-Fe_2_O_3_@Pt, Fe concentration: 200 ppm, Pt concentration: 20 ppm). After incubation for another 4 h, the cells were irradiated by ultrasound. Then the cells were stained with DCFH-DA probes and observed by CLSM and flow cytometry.

To further investigate the cytotoxicity of α-Fe_2_O_3_@Pt, the live and dead assay using typical calcein-AM/PI staining was carried out. Briefly, after seeding for 12–16 h, the cells were mixed with the as-prepared solutions (control, Pt NPs, α-Fe_2_O_3_, α-Fe_2_O_3_@Pt, Fe concentration: 200 ppm, Pt concentration: 20 ppm) and incubated for 4 h. Then the culture dishes were treated with or without US. After culturing for another 24 h, the culture medium was replaced with fresh medium containing calcein-AM (4 μmol L^−1^) and PI (8 μmol L^−1^). The fluorescence images were observed by CLSM under blue light excitation.

A typical oxygen indicator, [Ru(dpp)_3_] Cl_2_ (RDPP) was used to detect intracellular oxygen level [31]. Briefly, after seeded for 12–16 h, the cells were mixed with the as-prepared solutions (control, Pt NPs, α-Fe_2_O_3_, α-Fe_2_O_3_@Pt, Fe concentration: 200 ppm, Pt concentration: 20 ppm) and incubated for 4 h. Then the culture dishes were treated with or without US. After culturing for another 24 h, the medium was replaced with fresh medium containing RDPP (5 μM). The RDPP fluorescence images were observed by CLSM under blue light excitation.

To further verify the SDT effect of α-Fe_2_O_3_@Pt, the cell apoptosis analysis was measured by flow cytometry. The cells were treated in a similar way as live and dead cell staining assay. The cells were cultured for another 12 h after US irradiation and stained with annexin V (5 µL) and PI (5 µL) for 15 and 5 min, respectively. The cell apoptosis under different treatments was detected by the flow cytometer.

### In vivo study

All animal experiments were approved by the Ethics Committee of Sir Run Run Shaw Hospital. Healthy 4-week old female BALB/c nude mice were purchased from Shanghai Laboratory Animal Center and injected with 4T1 cells in the left side abdomen.

To study the US imaging property of FP NPs, contrast enhanced ultrasound (CEUS) imaging technique was used both in vitro and in vivo. A GE LOGIQ 9 unit (GE Healthcare, Waukesha, USA) and linear probe (9 L) were used and regular microbubble contrast agent was replaced by FP NPs. For the in vitro study, 100 mM of 30% H_2_O_2_ was mixed with FP solution (50 μg mL^−1^) and the bubble generation were recorded after 5 min by camera. Besides, the in vitro US images of H_2_O_2_ (10 mM), FP (50 μg mL^−1^), and FP/H_2_O_2_ mixture in centrifuge tubes were recorded by the probe, respectively. For the in vivo imaging, FP NPs (16 mg mL^−1^, 50 μL) were intratumorally (i.t.) injected into the tumor on mice and the images and curves were recorded at room temperature.

The tumor-bearing mice were divided randomly into seven groups (n = 5 per group) as follows to evaluate the SDT efficacy of FP NPs: (1) normal saline (NS, as control); (2) FP NPs (intravenous (i.v.) injection, 40 mg kg^−1^ based on Fe); (3) only US (1.0 MHz, 2.0 W cm^−2^, 3 min); (4) Fe_2_O_3_ + US (i.v. injection, 40 mg kg^−1^ based on Fe); (5) Pt NPs + US (i.v. injection, 4 mg kg^−1^); (6) Fe_2_O_3_/Pt + US (i.v. injection, Fe_2_O_3_ was simply mixed with Pt NPs); (7) FP NPs + US. At 24 h and 72 h after the i.v. injection, the tumors in the relevant groups were treated with US irradiation. The same injection and irradiation procedure were repeated on Day 5 and Day 10. The US irradiation was only focused on the tumor sites and the transducer was set at 1 cm above the tumor. In the meanwhile, the body weight and tumor volume were recorded every 2 days according to the following formula: width^2^ × length/2. After 14 days, the mice were sacrificed and the subcutaneous tumors were peeled off and weighed. The tumors were also incubated with anti-HIF-1α antibody overnight at 4 °C and were also treated with 4% paraformaldehyde solution for histopathological analysis including hematoxylin and eosin (H&E) staining and Ki-67 staining.

### Biosafety assessment

To verify the biosafety of FP NPs, the major organs (heart, liver, spleen, lung, and kidney) were collected on day 14 after different treatments for histopathological analysis. The fresh tissue samples were fixed in 4% paraformaldehyde solution overnight and stained with H&E subsequently. The blood samples at day 1, day 3 and day 7 were also collected for blood biochemistry assay and blood routine examination. To study the biodistribution of FP NPs, tissue samples (heart, liver, spleen, lung, kidney and tumor with 3 parallel) were collected at 0 h, 12 h, 24 h and 72 h after i.v. injection of FP NPs. The samples were digested with nitric acid/perchloric acid (9:1) at 255 ℃. The concentration of Pt was measured by ICP-OES analysis. Besides, the blood samples were also collected at 0.09, 0.25, 0.5, 1, 2, 6, 12 h for blood circulation assay.

## Results and discussion

### ***Synthesis and characterization of ***α***-Fe***_***2***_***O***_***3***_***@Pt***

**Fig. 2:**
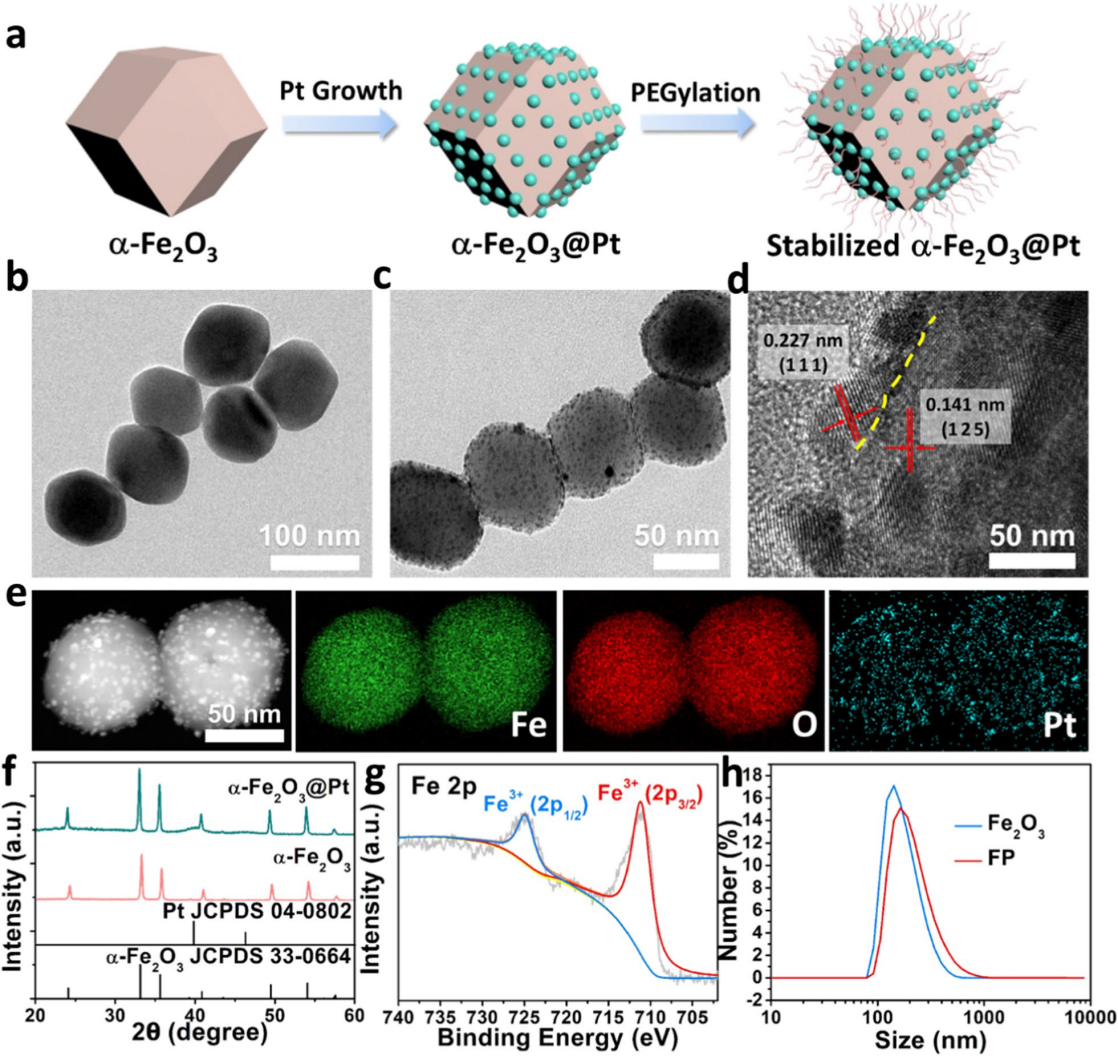
Synthesis of α-Fe_2_O_3_@Pt nanoparticles (FP NPs). **a** Scheme for the synthesis approach. TEM images of **b** α-Fe_2_O_3_ and **c** FP NPs. **d** High-resolution TEM image and **e** element mappings of FP NPs. **f** XRD patterns for α-Fe_2_O_3_ and FP NPs. **g** XPS spectra of the Fe 2p peaks in FP NPs. **h** The diameter distribution of Fe_2_O_3_ and FP NPs

In this study, α-Fe_2_O_3_ nanoparticles were prepared via a modified one-pot hydrothermal method, and subsequently mixed with prerequisite H_2_PtCl_6_ solution for the in situ growth of Pt nanocrystals at the surface. The as-prepared α-Fe_2_O_3_@Pt nanoparticles were further modified with mPEG-SH (FP NPs) (Fig. 2a). The as-prepared α-Fe_2_O_3_ NPs present uniform polyhedron structure with a mean diameter of 80–90 nm (Fig. 2b and Additional file 1: Fig. S1a). The Pt NPs with a diameter of ~ 10 nm distribute uniformly on the surface of FP NPs (Fig. 2c and Additional file 1: Fig. S1b). The high-resolution TEM image shows the typical lattice fringes of Pt nanocrystals and α-Fe_2_O_3_ matrix (Fig. 2d). The lattice spacing of 0.227 nm and 0.141 nm corresponds well to the (111) lattice plane of Pt NPs (JCPDS 04–0802) and the (125) lattice plane of hematite α-Fe_2_O_3_ (JCPDS 33–0664) (Fig. 2f), respectively. The yellow dash line in Fig. 1d indicates the interface between Pt and α-Fe_2_O_3_, confirming the formation of heterostructure. The weak intensity of the broad peak between 39.8° and 46.2° is attributed to the low proportion of Pt compared to α-Fe_2_O_3_ matrix. The element mapping images indicate that FP NPs contains Fe, O and Pt elements, and Pt presents uniformly across the entire particle (Fig. 2e). Furthermore, the X-ray photoelectron spectroscopy (XPS) spectra of FP NPs present typical trivalent Fe^3+^ peaks (Fig. 2g). With the surface integration of Pt nanocrystals, the particle hydrolyzed diameter increases from ~ 178 nm to ~ 222 nm (Fig. 2h). The clear Tyndall effect of α-Fe_2_O_3_ and FP NPs solutions was observed, indicating the particle stability (Additional file 1: Fig. S2a). From the Fourier transform infrared (FTIR) spectra, the peak of 1470 cm^−1^ in the spectrum of modified FP NPs corresponds to the bending vibration of -CH_2_- in mPEG-SH, verifying the successful anchoring of mPEG-SH (Additional file 1: Fig. S3). After PEGylation procedure, the FP NPs present excellent stability in the physiological solutions, including water, phosphate buffered saline (PBS), and RPMI 1640 medium (Additional file 1: Fig. S2b). The proportion of Fe and Pt in FP NPs is approximately 10:1 as measured via inductively coupled plasma mass spectrometry (ICP-OES).

### Oxygen production and US-triggered ROS induction

**Fig. 3:**
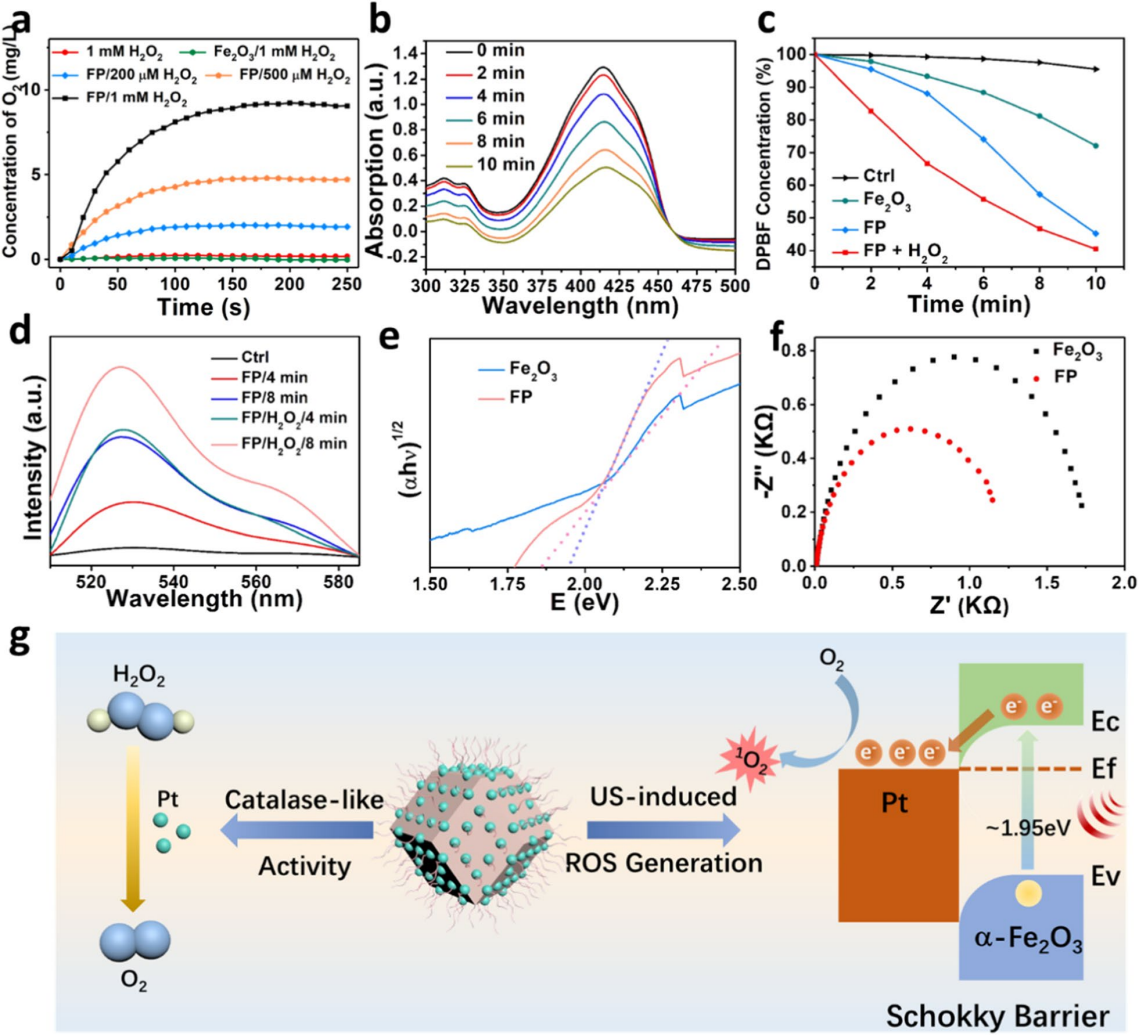
Characteristics of FP NPs. **a** The ability of O_2_ generation in the different concentrations of H_2_O_2_ for α-Fe_2_O_3_ and FP. **b** The UV–vis spectra of time-dependent DPBF degradation after incubated with FP under US irradiation (1.0 MHz, 1.0 W cm^−2^). **c** The absorption of DPBF after incubated with α -Fe_2_O_3_, FP, FP + H_2_O_2_ under US irradiation. **d** Time-dependent fluorescence intensities of SOSG after incubated with FP and FP + H_2_O_2_ under US irradiation. **e** Solid UV–vis spectra of α-Fe_2_O_3_ and FP. **f** Nyquist plots of α-Fe_2_O_3_ and FP obtained from EIS measurements. **g** Schematic illustration for the function mechanisms of FP NPs

Noble metal Pt presents intrinsic catalase-like activity, enabling the catalysis of H_2_O_2_ and O_2_ generation [2, 23]. In this study, the functionality in oxygen generation of nanozyme FP NPs was examined by detecting the concentration of dissolved oxygen in the presence of H_2_O_2_. As shown in Fig. 3a, α-Fe_2_O_3_ NPs does not present enzyme-like activity as no oxygen generation can be detected. In comparison, FP NPs generate O_2_ rapidly when incubation with different concentrations of H_2_O_2_ (200 μΜ, 500 μΜ, 1 mM), and the velocity of O_2_ generation is highly dependent to the concentration of H_2_O_2_. Subsequently, a common ROS probe, 1,3­diphenylisobenzofuran (DPBF), was used to evaluate the SDT performance. When mixed with FP NPs and irradiated with different duration of ultrasound, the absorption of DPBF decreases conspicuously (Fig. 3b), indicating the effective ultrasound-triggered ROS generation of FP NPs. To verify the crucial role played by Pt in the ultrasound-induced ROS generation, the SDT performance of pure DPBF, α-Fe_2_O_3_ alone, FP NPs with and without the addition of H_2_O_2_ was investigated (Additional file 1: Fig. S4). The absorption in the solutions of control, Fe_2_O_3_, FP NPs and FP NPs + H_2_O_2_ declined by ~ 5%, ~ 28%, ~ 55% and ~ 60% after 10 min ultrasound agitation, respectively (Fig. 3c). The superior ROS induction of FP NPs, with addition of H_2_O_2_, is attributed to the self-suppled oxygen induced by Pt nanocrystals. The ability of ^1^O_2_ production in FP NPs under US irradiation was also characterized by fluorescent singlet oxygen sensor green (SOSG) probe (Fig. 3d). The fluorescent signal intensity at 530 nm of SOSG increases clearly in the presence of FP NPs, and more agitated increase can be detected when adding H_2_O_2_ or increasing the irradiation time, as expected.

To uncover the mechanism for the promoted sonodynamic effect of FP NPs, solid ultraviolet spectra were utilized to calculate the bandgap of α-Fe_2_O_3_ and FP NPs (Additional file 1: Fig. S5). The band gap of pristine α-Fe_2_O_3_ is ~ 1.95 eV. After the integration of Pt nanocrystals on the surface, the band gap of the hybrid FP NPs declines to ~ 1.83 eV (Fig. 3e). The reduced bandgap suggests that the hybrid can adsorb more energy and generate holes and electrons in a more active manner. The electrochemical impedance spectrum (EIS) was measured to evaluate the charge transfer capacity and carrier separation efficiency of α-Fe_2_O_3_ and FP NPs (Fig. 3f). The EIS Nyquist plots of FP NPs show smaller semicircle than that of α-Fe_2_O_3_. This implies that Pt nanocrystals can serve as a capture trap to capture the induced electrons and facilitate the separation of electrons (e^−^) and holes (h^+^), thus improving ROS generation. Overall, the mechanism of the enhanced SDT effect by FP NPs is clear. As demonstrated in Fig. 3g, α-Fe_2_O_3,_ as a n-type semiconductor with a narrow band-gap of ~ 1.95 eV [32], induces the separation of h^+^ and e^−^ when adsorbing the ultrasound energy. Pt domains loaded on the surface act as electron sinks. The hypothesis is supported by the fact that the work function of semiconductor α-Fe_2_O_3_ (5.4 eV) is lower than that of noble metal Pt (5.65 eV) [33]. Thus, the Fermi levels of α-Fe_2_O_3_ is higher than that of Pt, causing the electrons transfer from α-Fe_2_O_3_ to Pt according to the minimum energy principle. The charges accumulate on the semiconductor, forming the space electric field and a Schottky barrier. In our case, the hybrids promote the sono-induced electrons rapidly transferring from α-Fe_2_O_3_ and then trapped by Pt domains, suppressing the electron–hole recombination. In addition, the electrons trapped by Pt domains trigger the reduction of dissolved molecular oxygen, both in the solution and from the catalysis of H_2_O_2_, and produce reactive oxygen species, ^1^O_2_.

### In vitro study

**Fig. 4:**
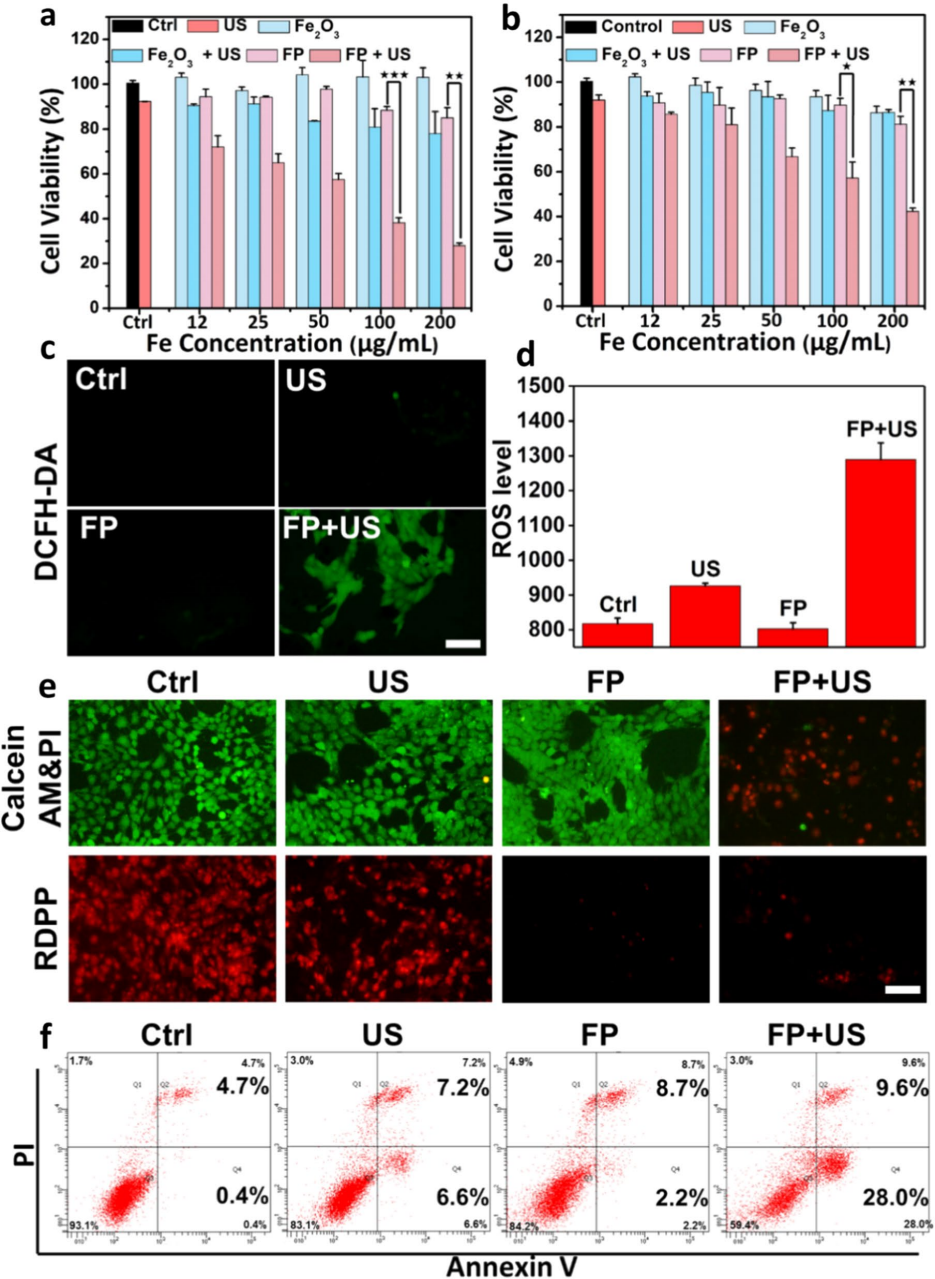
In vitro study. The relative cell viability of Fe_2_O_3_ and FP with or without US (1.0 MHz, 1.0 W m^−2^, 30 s) under **a** normoxic and **b** hypoxic conditions (***p < 0.001, **p < 0.01 and *p < 0.05). **c** The fluorescence ROS images and **d** quantitative analysis by flow cytometer of 4T1 cells staining with DCFH-DA and treated by FP NPs under different conditions. Scale bar: 40 μm. **e** The fluorescence images of 4T1 cells staining with calcein AM (green, live cells) & PI (red, dead cells) and RDPP and treated by FP NPs under the different conditions. Scale bar: 40 μm. **f** The flow cytometer apoptosis assay of 4T1 cells staining with PI and Annexin-FTIC

The cytotoxicity of FP NPs in normoxic and hypoxic condition was initially examined by cell counting kit-8 (CCK-8) assay (Fig. 4a and b). The sole US irradiation is harmless to the cells under both normoxic and hypoxic conditions. In the absence of US, Fe_2_O_3_ and FP NPs does not exhibit clear toxicity to 4T1 murine cancer cells, even at a high concentration (200 μg mL^−1^). When exposed to US (1.0 MHz, 1.0 W cm^−2^, 30 s) at normoxic condition, the cell viability in Fe_2_O_3_ group is gently decreased, by only ~ 20% at the concentration of 200 μg mL^−1^ (Fig. 4a). However, the survival rate of 4T1 cells evidently decreases when culturing with FP NPs after US irradiation, and only ~ 25% of cells survive when Fe concentration reaches 200 μg mL^−1^. Under a hypoxic condition mimicking tumor tissue microenvironment, the inhibition effect to tumor cells in different sample groups is weakened by different magnitude (Fig. 4b), as 4T1 cells are generally more resistant to stimulus interference in hypoxic condition. The antitumor effect of FP NPs is stronger than that of Fe_2_O_3_ due to its promoted ROS generation under ultrasound irradiation, as expected. The killing effect of FP NPs (200 μg mL^−1^) to 4T1 cells was verified by staining the dead and live cells with propidium iodide (PI) and calcein acetoxymethyl ester (calcein-AM), respectively (Fig. 4c). The fluorescence images demonstrated that pure FP NPs or US treatment alone does not present clear inhibition effect to 4T1 cells. When treated by FP NPs + US, most of cells exhibited significant red fluorescence, indicating the severe cell death induced by the SDT effect of FP NPs. The flow cytometry apoptosis assay in different protocols was also conducted to verify the synergistic therapeutic effect using the Annexin V-fluorescein isothiocyanate (Annexin-FTIC) and PI double staining principle (Fig. 4d), confirming that the majority of cells were killed after FP NPs + US treatment.

To reveal the mechanism of killing effect from FP NPs, the intracellular ROS production and oxygen content were examined. An oxidation-sensitive probe 2′,7′-dichloroflurescein diacetate (DCFH-DA), which could be oxidized to dichlorofluorescein (DCF) and emit green fluorescence in the presence of ROS, was used to determine the intracellular ROS generation in 4T1 cells [34]. No clear green fluorescence of DCF can be observed in the control group and the cells treated with sole FP NPs, which indicates that ROS generation in these groups is negligible (Fig. 4e). However, when irradiated with US, the cells show clear green fluorescence (Fig. 4e and Additional file 1: Fig. S8). And the cells treated with FP NPs + US displayed the most intense green fluorescence compared to all other groups, indicating its strongest ROS production. Furthermore, flow cytometry apoptosis assay was conducted to quantitatively evaluate the ROS production in different protocols (Fig. 4f and Additional file 1: Fig. S7). The results indicated that the order of intracellular ROS content is FP + US > US > FP and Control group, and the FP + US group displays a considerably increased intensity of fluorescence, which is consistent with the sign of fluorescence images. The intracellular oxygen content were examined by using a typical oxygen probe [Ru(dpp)_3_]Cl_2_ (RDPP), whose fluorescence could be quenched by O_2_ [31]. As shown in Fig. 4e, the cells of control group present strong red fluorescence of RDPP owing to the intrinsic hypoxia in tumor cells. The FP NPs group shows a weakened fluorescence due to the nanozyme activity of Pt. The cells treated by FP NPs with US display stronger fluorescence than that without US, which can be attributed to the transformation of O_2_ to ^1^O_2_ in the SDT process. As a comparison, pure Pt nanocrystals with the same size to that on the surface of FP NPs were synthesized (Additional file 1: Fig. S6). ROS production, live and dead and oxygen content of cells staining with DCFH-DA, calcein-AM/PI and RDPP after different treatments are shown in Additional file 1: Fig. S8. Pure Pt can act as a nanozyme to generate considerable amount of oxygen, while Fe_2_O_3_ could consume oxygen and generate ROS under US irradiation. All these phenomena indicate that FP NPs could act as a potential sonosensitizer with strong ameliorating property to hypoxic tumor microenvironment by combining the catalases-like and ultrasonic catalytic capabilities induced by the Schottky barrier formed between Pt and Fe_2_O_3_.

### In vivo antitumor efficacy and ultrasound imaging

**Fig. 5:**
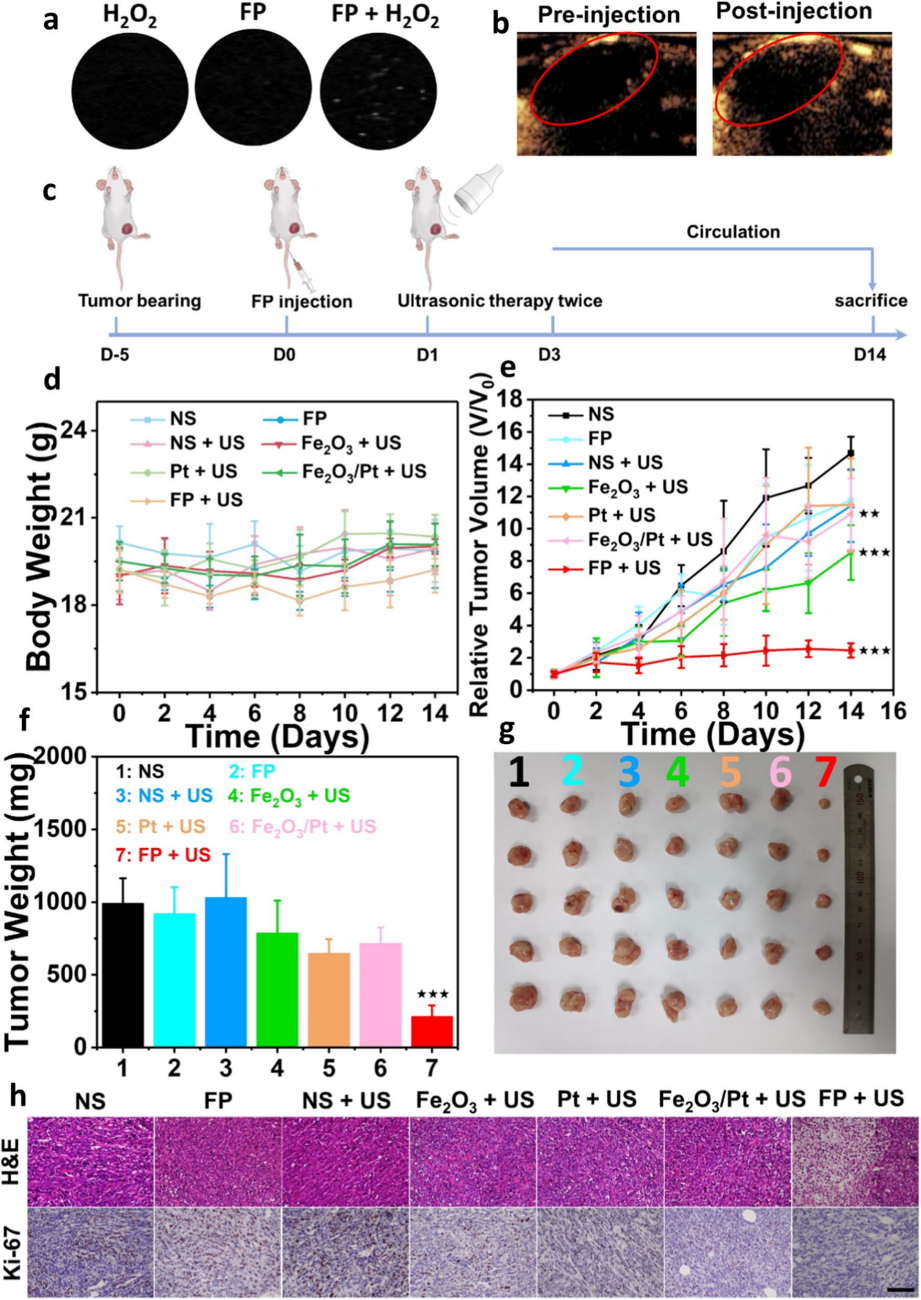
Tumor Inhibition and US imaging. **a** US imaging of H_2_O_2_, FP NPs and FP + H_2_O_2_. **b** The ultrasound contrast images of tumor before and after injection of FP NPs. **c** Experimental flow chart of in vivo study. The variations of **d** body weight and **e** relative tumor volume of mice from different groups (*n* = 5, mean ± SD). **f** Tumor weights (*n* = 5, mean ± SD) and **g** images from each groups after sacrificing the mice on the 14th day. (h) H&E and Ki-67 staining of tumor tissues from the mice in different groups (scale bar: 100 μm). ***p < 0.001, **p < 0.01 and *p < 0.05

In general, gas bubbles may induce certain reflection to ultrasound irradiation due to the density difference between tissue and gas [35]. In this study, considerable amount of O_2_ bubbles is generated when FP NPs are added into H_2_O_2_ solution, while H_2_O_2_ or FP NPs solutions barely induce bubbles (Additional file 1: Fig. S9a). As a result, the tube filled with FP/H_2_O_2_ mixture shows strong dotted echoes in B-mode ultrasound image, which is highly distinctive comparing to the other two samples (Fig. 5a), implying FP NPs may serve as a US contrast agent (UCA). To verify its potential characteristics favoring the in vivo US imaging, FP NPs were intratumorally injected on mice, and interestingly a distinct variation in US contrast was observed at the tumor site after a certain period of time (Fig. 5b). The time-intensity dependence analysis shows a gradual and clear rise in the US signal in the region containing FP NPs, while that without FP NPs maintains the original contrast (Additional file 1: Fig. S9b).

The in vivo study, revealing the antitumoral properties of FP NPs, was conducted on 4T1 tumor-bearing mouse model. The mice with the initial tumor volume of 100 mm^3^ were used and randomly divided into 7 groups (*n* = 5): (1) Normal saline (NS, as control); (2) FP NPs (intravenous injection (i.v.), 40 mg kg^−1^ based on Fe); (3) only US (1.0 MHZ, 2.0 W cm^−2^, 3 min); (4) Fe_2_O_3_ + US (i.v. injection, 40 mg kg^−1^ based on Fe); (5) Pt NPs + US (i.v. injection, 4 mg kg^−1^); (6) Fe_2_O_3_/Pt + US (i.v. injection, Fe_2_O_3_ was simply mixed with Pt NPs); (7) FP NPs + US. At 24 h after i.v. injection, US irradiation was conducted in Group 3 to 7, and the operation was repeated for three times on Day 0, Day 5 and Day 10, respectively (Fig. 5c). After 2 weeks’ treatment, the body weights of mice in all groups showed no clear difference (Fig. 5d), while the tumor volume and weight presented considerable difference (Fig. 5e–f). The tumors in the control group (NS) showed the most rapid proliferation, and the tumor growth in Group 2 and 3 was postponed by a marginal magnitude. Compared to sole treatment by Fe_2_O_3_, Pt or the mixture, Group 7 (FP + US) presented the strongest inhibition to tumor growth, and the inhibition rate reached as high as ~ 78%. The representative photographs of tumor-bearing mice and tumors peeled off also validate the findings (Fig. 5g and Additional file 1: Fig. S10). In addition, the tumors collected from all groups were stained using H&E and Ki-67 for the histological examinations (Fig. 5h). Severe vacuolization and nuclear pyknosis, which are the typical apoptotic characteristics, were observed by the H&E stained images in FP + US group, indicating notable cellular damage of tumor tissue. The markedly decreased number of Ki-67 positive cells was observed in the FP + US group, comparing to other groups, suggesting its clear inhibition to tumor proliferation, as expected. In addition, to investigate the in vivo hypoxia relieving phenomenon of FP NPs, the immunohistochemistry assay using anti-HIF-1α antibody was conducted on tumor slices following the standard protocol. The results reveal that compared with groups treated with Fe_2_O_3_ + US and normal saline, groups treated with FP NPs, FP NPs + US, Pt + US and Fe_2_O_3_/Pt + US exhibited significantly reduced HIF-1α expressions. This indicate that the tumor hypoxia can be effectively relived by the functional characteristic of Pt in O_2_ generation (Additional file 1: Fig. S11).

**Fig. 6:**
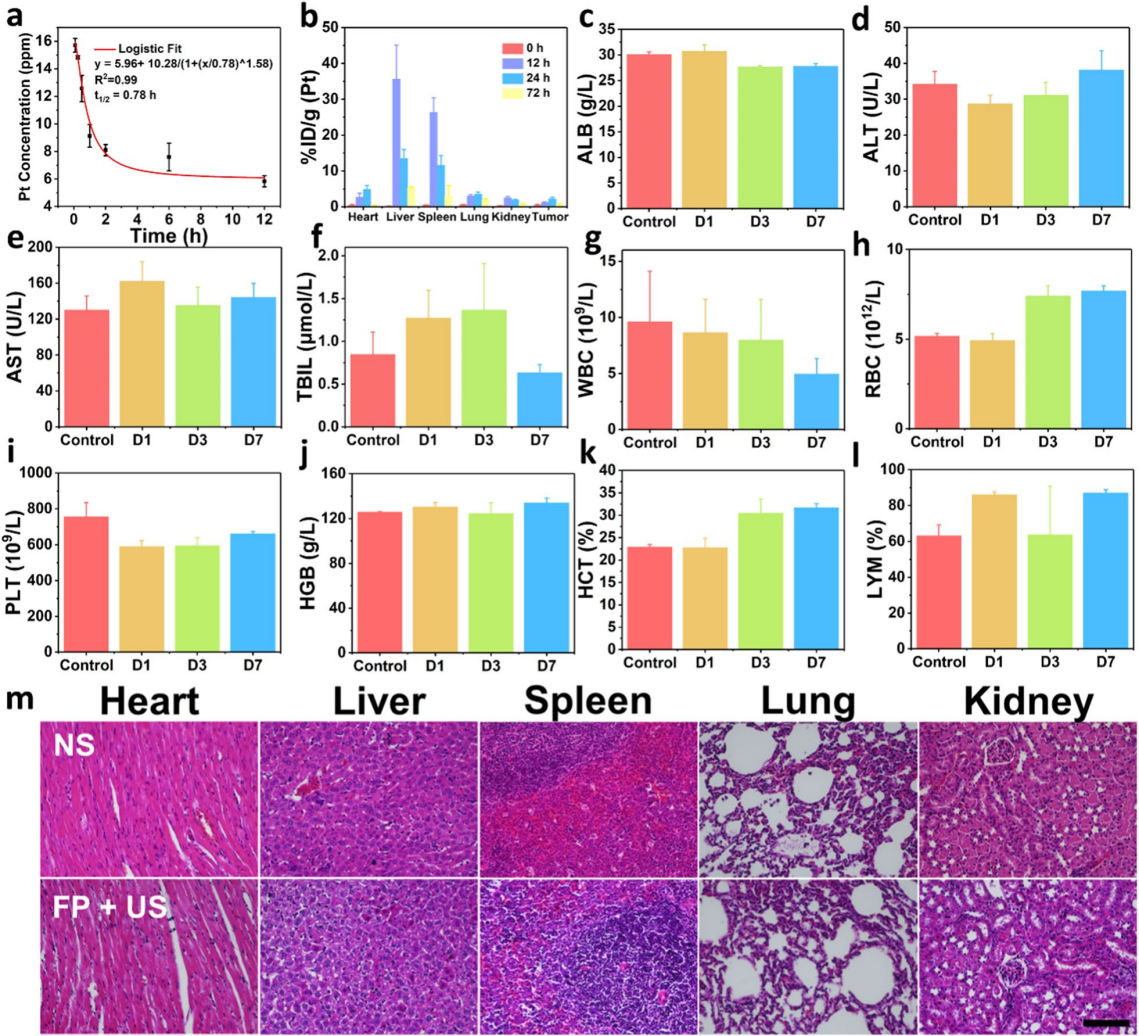
In vivo biodistribution and biosafety. **a** Blood clearance and blood half-life in mice (*n* = 3, mean ± SD) after i.v. injection of FP NPs at the different time points. **b** Biodistribution of Pt in mice (*n* = 3, mean ± SD) after i.v. injection of FP NPs at the different time points. **c**–**l** Blood biochemistry and hematological analysis of mice after i.v. injection of FP NPs at the different time points (day 1, day 3 and day 7) including serum albumin (ALB), alanine aminotransferase (ALT), aspartate aminotransferase (AST), total bilirubin (TBIL), white blood cell (WBC), red blood cell (RBC), blood platelet (PLT), hemoglobin (HGB), hematocrit (HCT) and lymphocytes ratio (%LYM) recorded on day 1, day 3 and day 7 (*n* = 3, mean ± SD). **m** H&E staining of the major organs (heart, liver, spleen, lung and kidney) of mice to examine the pathological changes treated with NS (control) and FP + US groups. Scale bar:100 μm

The biosafety of FP NPs was investigated subsequently. The blood clearance and long-term biodistribution of FP NPs at different time points were assessed by ICP-OES analysis (Fig. 6a, b). The blood circulation half-life of FP NPs is ~ 0.78 h, and the particles tend to accumulate in tumor, liver and spleen after intravenous injection. At 72 h after injection, the concentration of FP NPs in liver and spleen decreases due to metabolism and excretion. Moreover, the blood biochemistry and hematologic analysis were carried out after intravenous injection of FP NPs for different periods (day1, day 3 and day 7) (Fig. 6c–l and Additional file 1: Fig. S12). No abnormalities are detected in all main indices, including serum albumin (ALB), alanine aminotransferase (ALT), aspartate aminotransferase (AST), total bilirubin (TBIL), white blood cell (WBC), red blood cell (RBC), blood platelet (PLT), hemoglobin (HGB), hematocrit (HCT), lymphocytes ratio (LYM), mean corpuscular volume (MCV), mean corpuscular hemoglobin concentration (MCHC) and mean corpuscular hemoglobin (MCH). This suggests that the injection of FP NPs does not induced clear negative effect to the animal system. As demonstrated by the H&E staining images, no visible difference and damage can be observed in major organs (heart, liver, spleen, lung and kidney) of all groups after treatment, suggesting that FP NPs do not present apparent histological toxicology (Fig. 6m and Additional file 1: Fig. S13).

## Conclusions

In this study, we have successfully designed and synthesized fine α-Fe_2_O_3_ nanoparticles armored with Pt nanocrystals (α-Fe_2_O_3_@Pt) for sonodynamic therapy with imaging-guidance and self-Supplied O_2_. In this system, the pairing of α-Fe_2_O_3_ and noble metal Pt forms an effective capture trap for electrons, preventing the recombination of ultrasound-induced electrons and holes. The unique heterostructure constructed in this hybrid system, serving as a Schokky barrier, drastically promotes the induction of toxic ^1^O_2_ under the agitation of ultrasound. Meanwhile, Pt nanocrystals integrated at the surface of α-Fe_2_O_3_@Pt act as an effective nanozyme to trigger the decomposition of H_2_O_2_ and generate considerable amount of O_2_. The blooming O_2_ induction may in turn modulate the tumor hypoxia, favoring the efficacy of SDT, and enable a clear contrast enhancement to ultrasound signals. In consequence, α-Fe_2_O_3_@Pt appears to induce effective tumor inhibition with imaging guidance, both in vitro and in vivo, while presenting expected biosafety. This study has therefore demonstrated a highly potential platform for ultrasound-driven tumor theranostic, and may inspire further explorations in nanomedicine with more rational material design.

## Supplementary Information

**Additional file 1: Figure S1.** SEM images of (a) Fe_2_O_3_ and (b) FP NPs. **Figure S2**. Colloidal stability of FP NPs. (a) Tyndall effect of Fe_2_O_3_ (left) and FP NPs (right). (b) DLS profiles and the optical images of FP NPs in water, PBS and serum (from left to right) after 4 h. **Figure S3.** The FTIR spectra of mPEG-SH, FP NPs and FP NPs modified with mPEG-SH. **Figure S4**. The UV–vis spectra of time-dependent DPBF degradation after incubation with (a) blank, (b) Fe_2_O_3_, (c) FP + H_2_O_2_ (200 μM) under US irradiation (1.0 MHZ, 1.0 W cm^−2^). **Figure S5**. The solid UV–vis spectra of Fe_2_O_3_ and FP NPs. **Figure S6**. TEM image of pure Pt NPs with the same size as that on the surface of FP NPs. **Figure S7**. ROS fluorescence intensity of 4T1 cells by flow cytometry analysis after incubation with FP NPs under different conditions. **Figure S8**. The fluorescence images of 4T1 cells staining with DCFH-DA, calcein AM (green, live cells)/ PI (red, dead cells) and RDPP after different treatments. **Figure S9.** (a) The optical images of O_2_ generation after incubation with H_2_O_2_ (100 mM), FP NPs and FP + H_2_O_2_ (100 mM) for 5 min. (b) In vivo US imaging and time-intensity curve analysis of contrast-enhanced ultrasound imaging after intratumoral injection of FP NPs. **Figure S10**. The representative mice photographs of each groups recorded every 2 days. **Figure S11.** The immunohistochemistry detection for HIF-1α of tumor tissues from the mice in different groups. **Figure S12.** Routine blood analysis including mean corpuscular volume (MCV), mean corpuscular hemoglobin concentration (MCHC) and mean corpuscular hemoglobin (MCH). **Figure S13.** H&E staining of the major organs (heart, liver, spleen, lung and kidney) of mice to examine the pathological changes treated with FP, Fe_2_O_3_ + US, Pt + US and Fe_2_O_3_/Pt + US.

## Data Availability

The data used and/or analysed to support the current study are available from the corresponding author on reasonable request.
