# Supplementary material for: α-Fe2O3@Pt heterostructure particles to enable sonodynamic therapy with self-supplied O2 and imaging-guidance
Source: J Nanobiotechnology. 2021 Nov 4;19:358. doi: 10.1186/s12951-021-01105-x (PMC8569996; doi:10.1186/s12951-021-01105-x)
Supplement: Supplementary file 1 — Additional file 1: Figure S1. SEM images of (a) Fe2O3 and (b) FP NPs. Figure S2. Colloidal stability of FP NPs. (a) Tyndall effect of Fe2O3 (left) and FP NPs (right). (b) DLS profiles and the optical images of FP NPs in water, PBS and serum (from left to right) after 4 h. Figure S3. The FTIR spectra of mPEG-SH, FP NPs and FP NPs modified with mPEG-SH. Figure S4. The UV–vis spectra of time-dependent DPBF degradation after incubation with (a) blank, (b) Fe2O3, (c) FP + H2O2 (200 μM) under US irradiation (1.0 MHZ, 1.0 W cm−2). Figure S5. The solid UV–vis spectra of Fe2O3 and FP NPs. Figure S6. TEM image of pure Pt NPs with the same size as that on the surface of FP NPs. Figure S7. ROS fluorescence intensity of 4T1 cells by flow cytometry analysis after incubation with FP NPs under different conditions. Figure S8. The fluorescence images of 4T1 cells staining with DCFH-DA, calcein AM (green, live cells)/ PI (red, dead cells) and RDPP after different treatments. Figure S9. (a) The optical images of O2 generation after incubation with H2O2 (100 mM), FP NPs and FP + H2O2 (100 mM) for 5 min. (b) In vivo US imaging and time-intensity curve analysis of contrast-enhanced ultrasound imaging after intratumoral injection of FP NPs. Figure S10. The representative mice photographs of each groups recorded every 2 days. Figure S11. The immunohistochemistry detection for HIF-1α of tumor tissues from the mice in different groups. Figure S12. Routine blood analysis including mean corpuscular volume (MCV), mean corpuscular hemoglobin concentration (MCHC) and mean corpuscular hemoglobin (MCH). Figure S13. H&E staining of the major organs (heart, liver, spleen, lung and kidney) of mice to examine the pathological changes treated with FP, Fe2O3 + US, Pt + US and Fe2O3/Pt + US. [file 12951_2021_1105_MOESM1_ESM.docx]

**Supporting Information**

**α-Fe_2_O_3_@Pt Heterostructure** **Particles to Enable Sonodynamic Therapy with Self-supplied O_2_ and Imaging-guidance**

*Tian Zhang* ^1 ¤^*, Qiang Zheng* ^2 ¤^, *Yike Fu* ^1,3 ¤^, *Congkun Xie* ^1^*, Gonglin Fan* ^2^*, Yifan Wang ^2^, Yongjun Wu* ^1^*, Xiujun Cai* ^2^*, Gaorong Han* ^1,^*, Xiang Li* ^1^*^,3^* *

^1^ State Key Laboratory of Silicon Materials, School of Materials Science and Engineering, Zhejiang University, Hangzhou, Zhejiang 310027, P. R. China

^2^ Key Laboratory of Endoscopic Technique Research of Zhejiang Province, Sir Run Run Shaw Hospital, Zhejiang University, Hangzhou 215123, P. R. China

^3^ ZJU-Hangzhou Global Scientific and Technological Innovation Center, Zhejiang University, Hangzhou, 311200, P.R. China.

^¤^ Authors with equal contribution

* Corresponding Author: xiang.li@zju.edu.cn (XL)

**
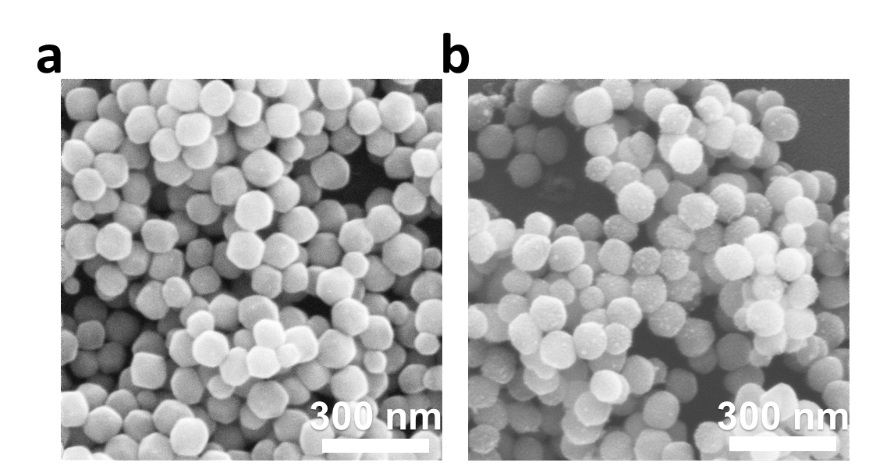
**

**Figure S1.** SEM images of (a) Fe_2_O_3_ and (b) FP NPs.

**
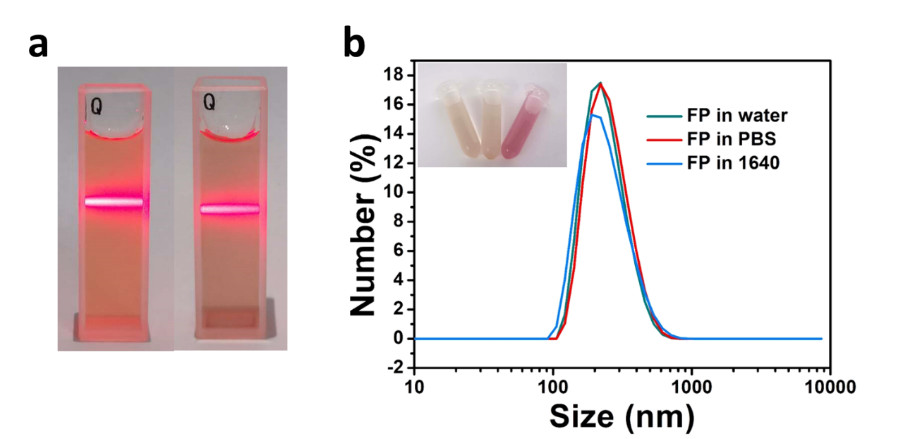
**

**Figure S2.** Colloidal stability of FP NPs. (a) Tyndall effect of Fe_2_O_3_ (left) and FP NPs (right). (b) DLS profiles and the optical images of FP NPs in water, PBS and serum (from left to right) after 4 h.


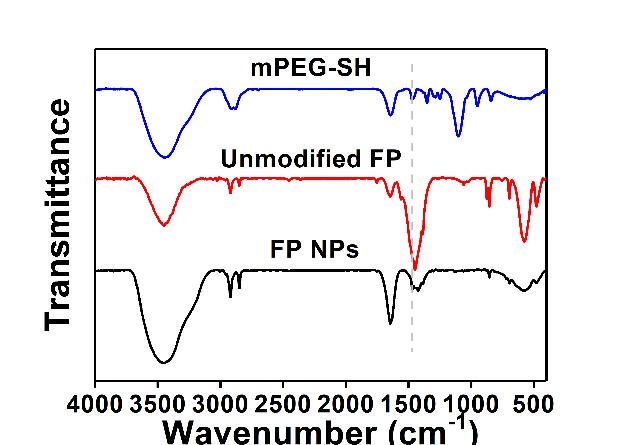


**Figure S3.** The FTIR spectra of mPEG-SH, FP NPs before and after modified with mPEG-SH. The peak of 1470 cm^-1^ in the spectrum of FP NPs could correspond to the bending vibration of -CH_2_- in mPEG-SH, which demonstrated the successful loading of mPEG-SH on FP NPs.

**
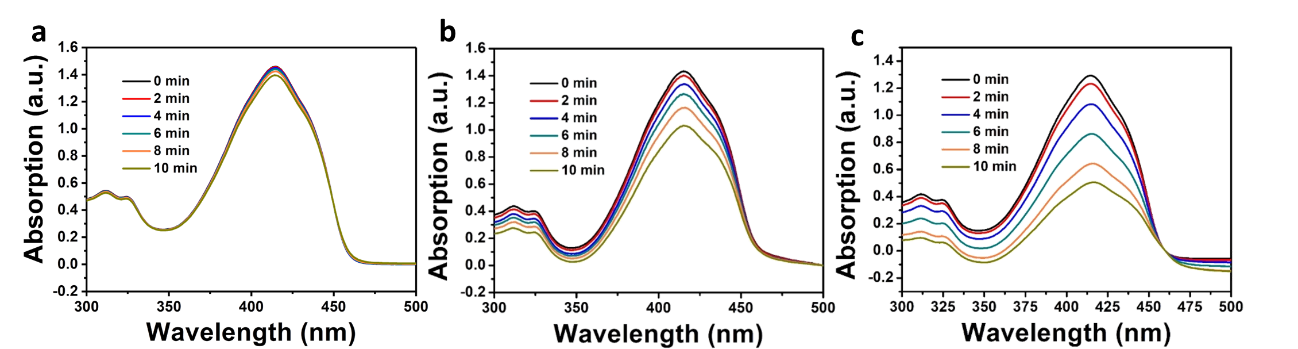
**

**Figure S4.** The UV-vis spectra of time-dependent DPBF degradation after incubation with (a) blank, (b) Fe_2_O_3_, (c) FP + H_2_O_2_ (200 μM) under US irradiation (1.0 MHZ, 1.0 W cm^-2^)

**
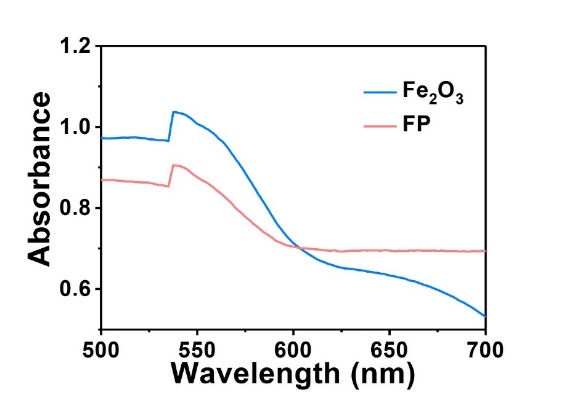
**

**Figure S5.** The solid UV-vis spectra of Fe_2_O_3_ and FP NPs.

**
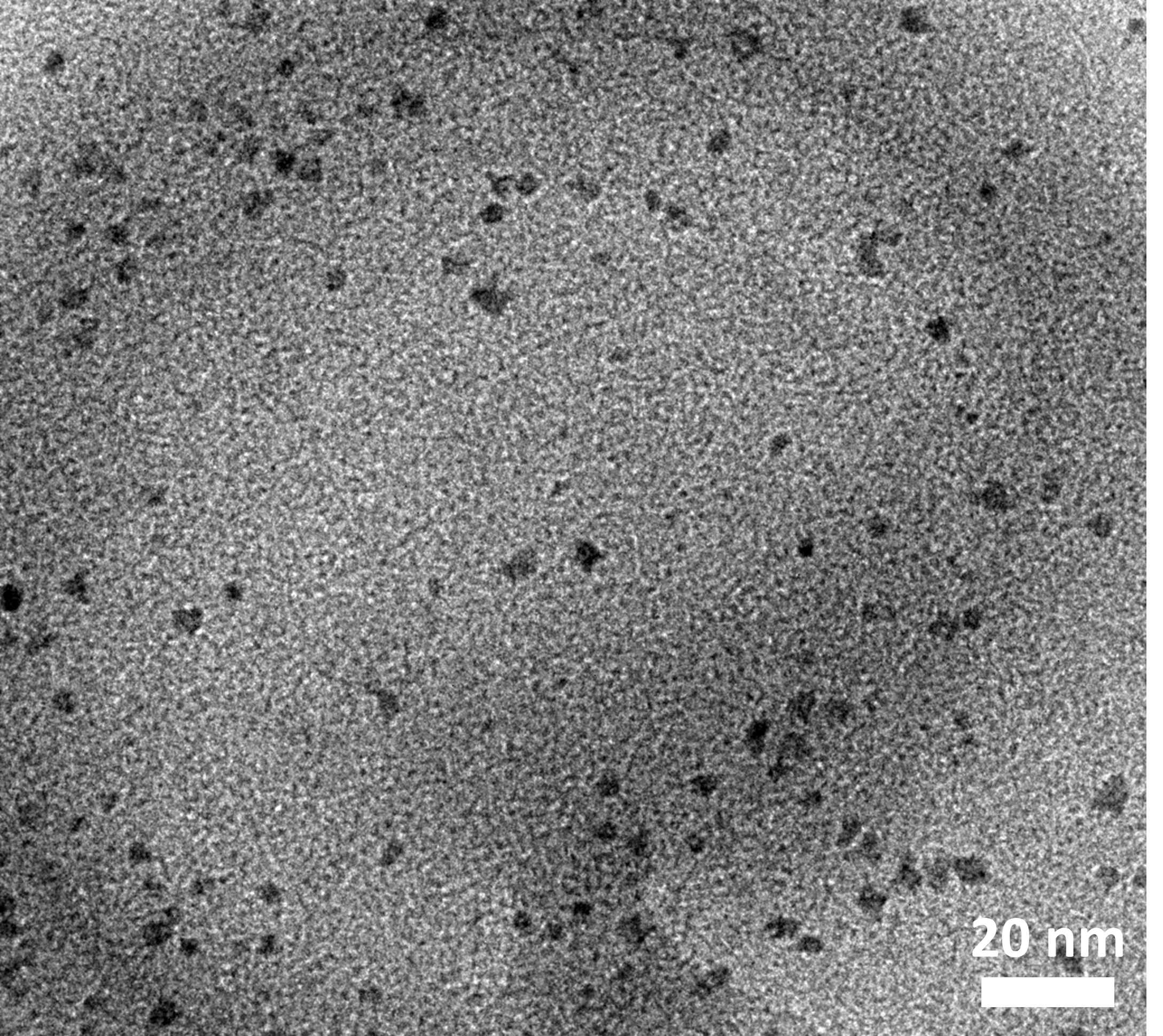
**

**Figure S6.** TEM image of pure Pt NPs with the same size as that on the surface of FP NPs.

**
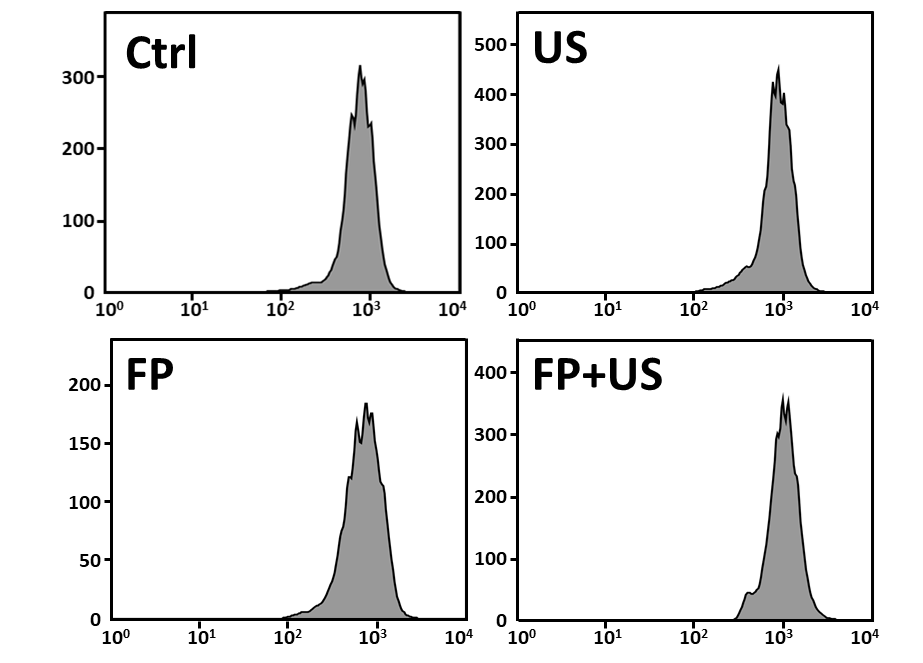
**

**Figure S7.** ROS fluorescence intensity of 4T1 cells by flow cytometry analysis after incubation with FP NPs under different conditions.

**
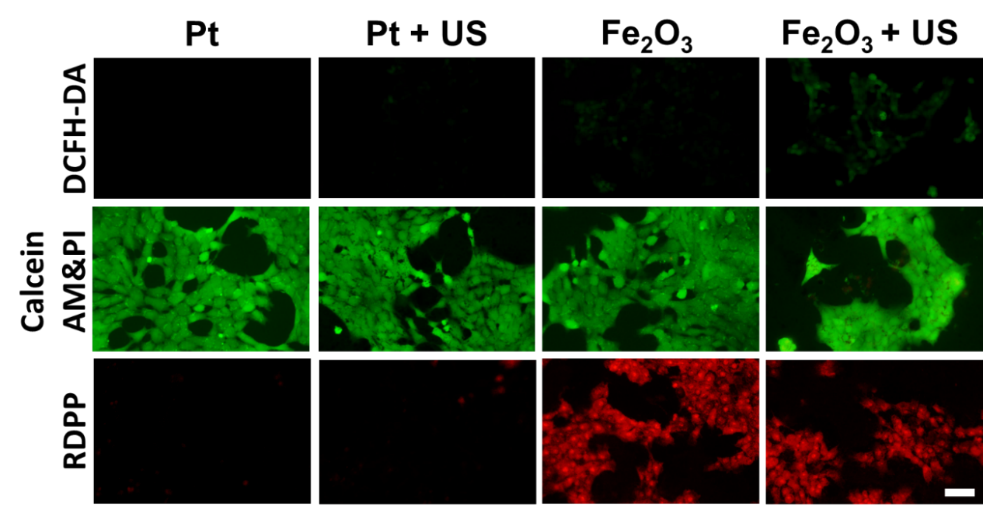
**

**Figure S8.** The fluorescence images of 4T1 cells staining with DCFH-DA, calcein AM (green, live cells)/ PI (red, dead cells) and RDPP after different treatments.

**
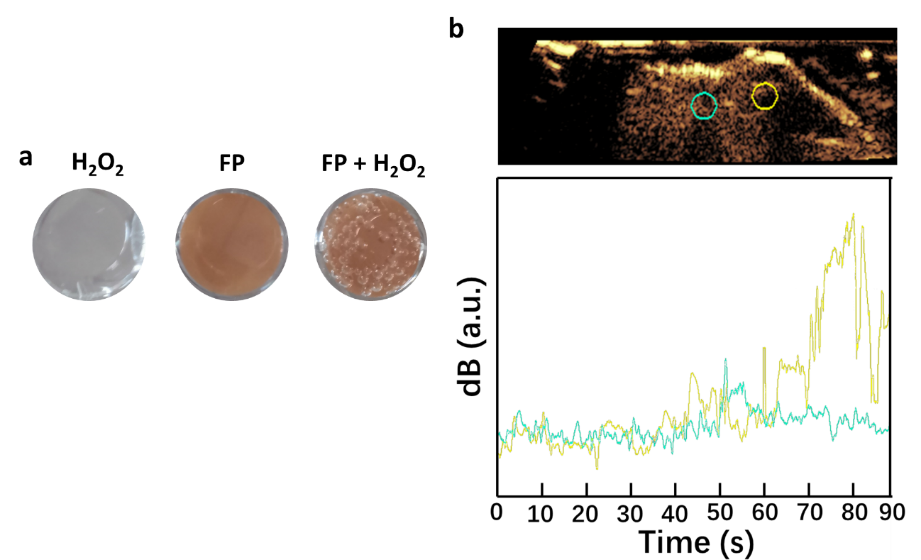
**

**Figure S9.** (a) The optical images of O_2_ generation after incubation with H_2_O_2_ (100mM), FP NPs and FP + H_2_O_2_ (100mM) for 5 minutes. (b) *In vivo* US imaging and time-intensity curve analysis of contrast-enhanced ultrasound imaging after intratumoral injection of FP NPs. The curves correspond to the intratumoral (yellow) and extratumoral (green) area, respectively.

**
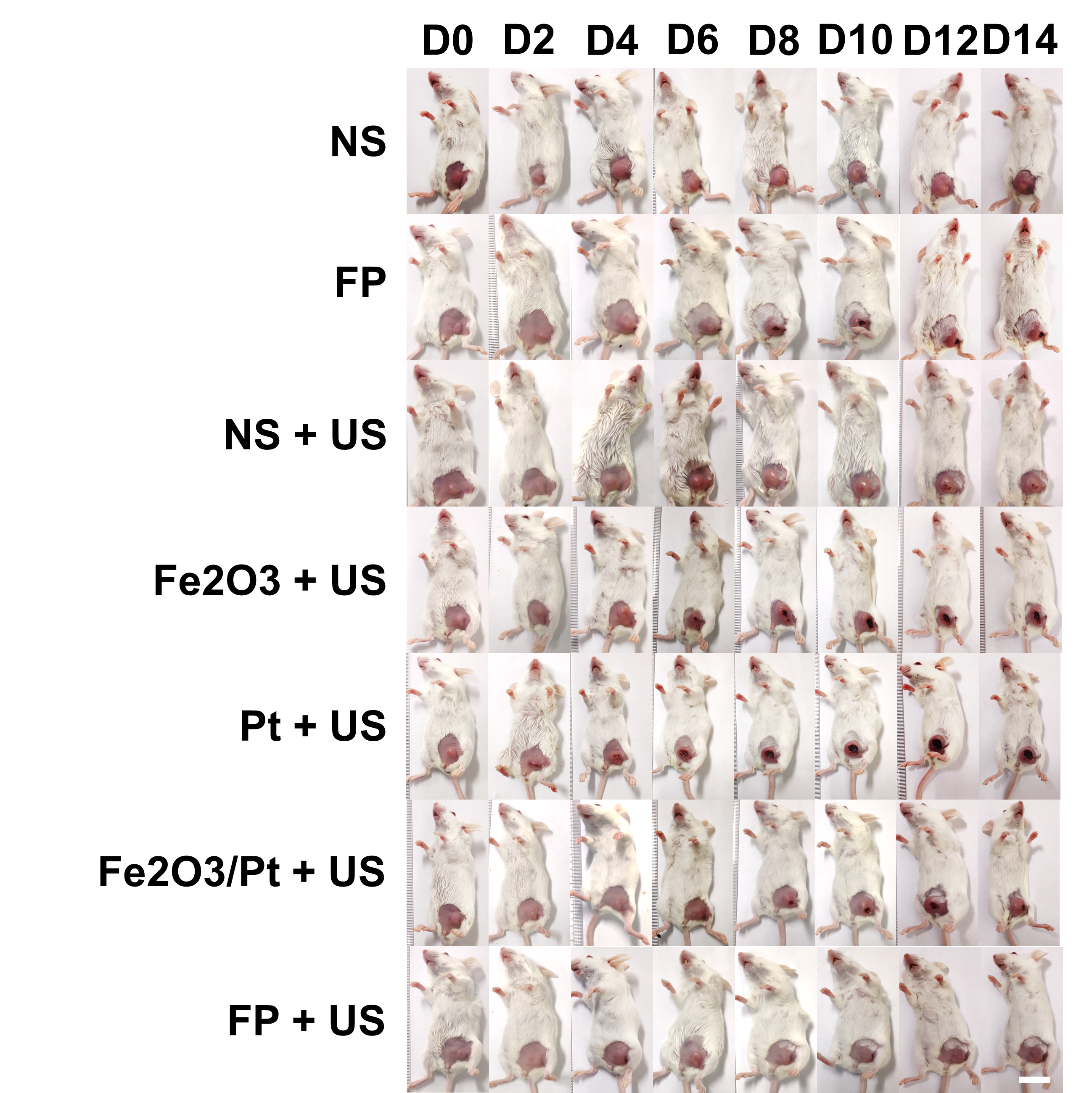
**

**Figure S10.** The representative mice photographs of each groups recorded every 2 days (scale bar = 2 cm). It reveals that FP + US group has excellent SDT efficacy for tumor growth inhibition and the mice of each groups have no obvious damage.


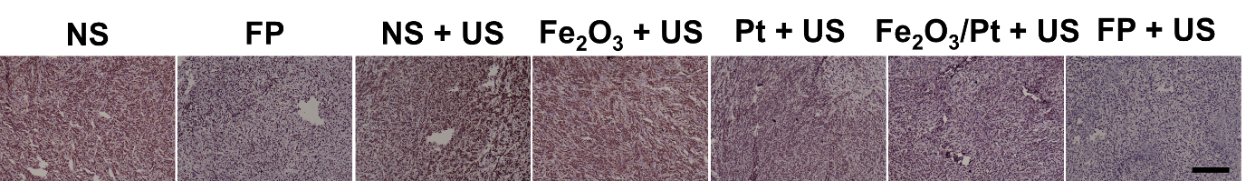


**Figure S11.** The immunohistochemistry detection for HIF-1α of tumor tissues from the mice in different groups (scale bar = 200 μm). It reveals that FP NPs and FP NPs + US exhibited obviously reduced HIF-1α expressions compared with control group.


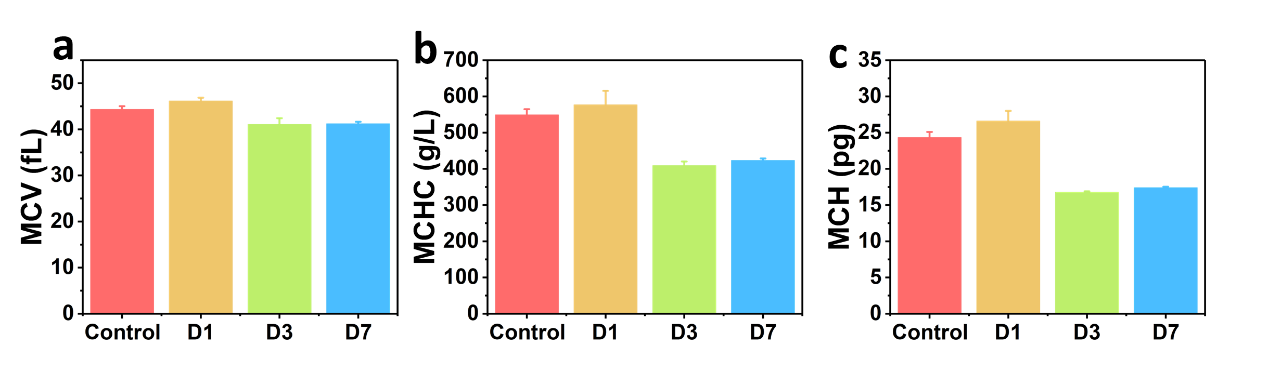


**Figure S12.** Routine blood analysis including mean corpuscular volume (MCV), mean corpuscular hemoglobin concentration (MCHC) and mean corpuscular hemoglobin (MCH).

**
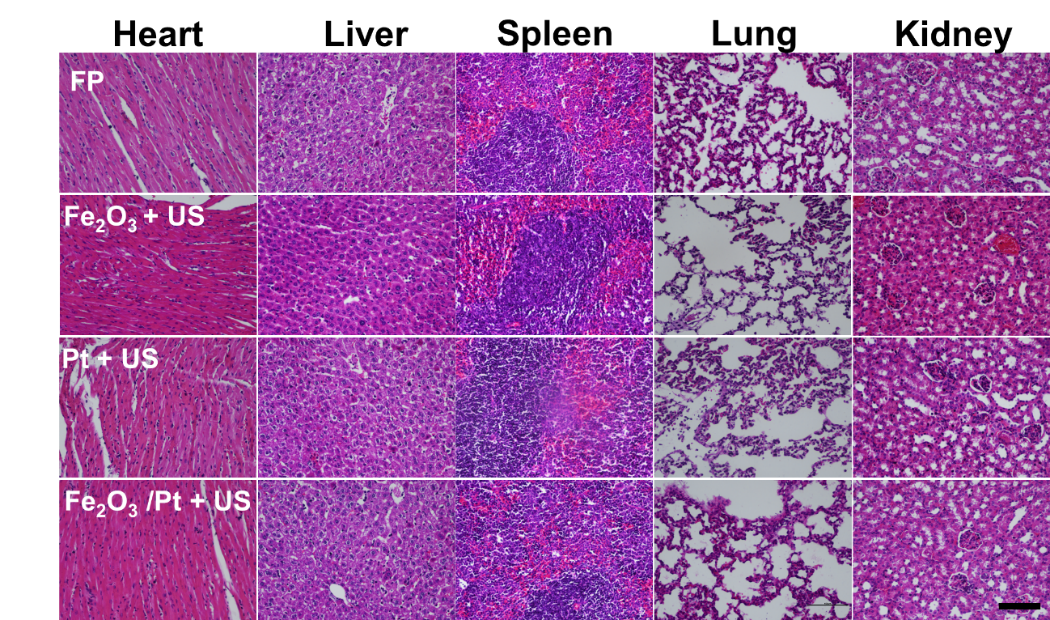
**

**Figure S13.** H&E staining of the major organs (heart, liver, spleen, lung and kidney) of mice to examine the pathological changes treated with FP, Fe_2_O_3_ + US, Pt + US and Fe_2_O_3_/Pt + US. Scale bar = 100 μm.
